# Teaching Recombinable Motifs Through Simple Examples

**DOI:** 10.1111/cogs.70103

**Published:** 2025-08-14

**Authors:** Huang Ham, Bonan Zhao, Thomas L. Griffiths, Natalia Vélez

**Affiliations:** ^1^ Department of Psychology Princeton University; ^2^ Institute for Language, Cognition and Computation, School of Informatics University of Edinburgh; ^3^ Department of Computer Science Princeton University

**Keywords:** Pedagogy, Social learning, Bayesian computational models, Cultural evolution

## Abstract

A hallmark of effective teaching is that it grants learners not just a collection of facts about the world, but also a toolkit of abstractions that can be applied to solve new problems. How do humans teach abstractions from examples? Here, we applied Bayesian models of pedagogy to a necklace‐building task where teachers create necklaces to teach a learner “motifs” that can be flexibly recombined to create new necklaces. In Experiment 1 (*N* = 151), we find that human teachers produce necklaces that are simpler (i.e., have lower algorithmic complexity) than would be expected by chance, as indexed by a model that samples uniformly from all necklaces that contain the target motifs. This tendency to select simpler examples is captured by a pedagogical sampling model that tries to maximize the learner's belief in the true motifs by prioritizing examples that have fewer alternative interpretations. In Experiment 2 (*N* = 295), we find that simplicity is beneficial. Human learners recover the underlying motifs better when teachers produce simpler sequences, as predicted by the pedagogical sampling model. However, humans learn best from human teachers rather than from model‐generated examples, which suggests that human teachers have additional expectations about how learners will interpret examples that are not captured by standard models of teaching. Our work provides a principled framework to understand when and why teachers use simple examples to convey abstract knowledge.

## 1 Introduction

Teaching equips learners with abstractions that can be applied to solve new problems and create new things. One important kind of abstraction is a *motif*, a recurring design element that can be flexibly combined into larger works. Motifs are ubiquitous in cultural products and often bear traces of the communities that produce them, such as the elaborate cable‐knit patterns of Aran sweaters, the fundamental rhythmic pattern or *clave* of salsa music, and meander designs on the borders of Greek pottery. These traces are not merely stylistic flourishes—they carry social meaning. Even young children can extract meaning from design elements, such as whether a design was passed down from one person to another (Jara‐Ettinger & Schachner, 2024; Pesowski, Quy, Lee, & Schachner, 2020).

Motifs exhibit two core characteristics of abstractions (Burgoon, Henderson, & Markman, 2013). First, motifs are not bound to specific objects, but can instead be abstracted away from particular instances to enable the creation of never‐before‐seen objects. For example, in knitting, the basic stitches (knits and purls) can be combined to form recurring motifs such as stockinette and rib stitch. A novice knitter who learns stockinette by making a dishcloth can then apply this motif to create endless new designs, including hats, scarves, sweaters, and blankets. Second, motifs expose regularities between different instances, and can thus make it easier to retain and communicate complex information (Mathy, Friedman, & Gauvrit, 2024; McCarthy, Hawkins, Holdaway, Wang, & Fan, 2021; Wu, Thalmann, & Schulz, 2025). For example, expert chess players recall meaningful board configurations more easily than random arrangements because their memories are organized around familiar motifs, such as openings and defensive positions (Chase & Simon, 1973; de Groot, 1978). Because motifs enable creative reuse and organize complex information, understanding how humans teach motifs provides a window into how abstract concepts are transmitted.

Teaching can take many forms, including verbal descriptions, demonstrations, feedback, and formal classroom instruction (Kline, 2015). In the present work, we focus on a minimal scenario in which teachers transmit motifs by providing a single, carefully crafted example. While this scenario is simpler than real‐world teaching, it allows us to isolate the factors that teachers prioritize when selecting information to communicate. Existing computational theories have formalized teaching from examples as a series of recursive, cooperative inferences: Teachers select examples that will maximize a learner's belief in a target concept, and learners work backwards from the examples provided to infer the concept that the teacher is trying to communicate to them (Gweon, 2021; Shafto, Goodman, & Griffiths, 2014; Shafto, Wang, & Wang, 2021). These *pedagogical sampling models* have been applied to study a wide variety of communicative behaviors, including how teaching through demonstration differs from goal‐directed behavior (Ho, Littman, MacGlashan, Cushman, & Austerweil, 2016; Tominaga, Knoblich, & Sebanz, 2022), how parents tune their speech to teach phonetic structures to infants (Eaves, Feldman, Griffiths, & Shafto, 2016), and how teachers improve their teaching based on feedback from learners (Chen, Palacci, Vélez, Hawkins, & Gershman, 2024). In addition, these computations appear to be neurally instantiated in brain regions implicated in social reasoning when teachers make decisions about what information to communicate to a learner (Vélez, Chen, Burke, Cushman, & Gershman, 2023). Thus, teaching from examples offers a controlled setting in which to study the basic computational principles that underlie effective teaching across social contexts and modalities.

Understanding how teachers transmit motifs poses new challenges for existing computational theories of teaching. Prior work has largely focused on capturing how learners acquire solutions to particular problems (such as how to operate a particular toy; Aboody, Velez‐Ginorio, Santos, & Jara‐Ettinger, 2023; Bridgers, Jara‐Ettinger, & Gweon, 2020; Buchsbaum, Gopnik, Griffiths, & Shafto, 2011) or identify the extension of particular categories (such as inferring the extent of a hidden shape on a canvas; Shafto et al., 2014; Vélez et al., 2023) from minimal examples. Most of these problems involve a teacher choosing examples that constitute a part of the target that they intend to teach, such as a single function of a toy with many functions or a single pixel inside a larger shape. Teaching motifs presents the converse problem. For example, suppose an expert knitter draws a novice's attention to the rib stitch pattern on the collar of a sweater. Her goal is not to help the novice identify sweaters based on this distinctive subcomponent, as is the case in traditional teaching games (Avrahami et al., 1997). Rather, the subcomponent *itself* is the target of teaching; the novice can later abstract away this subcomponent to knit sock cuffs and hatbands. Compared to traditional teaching problems, extracting reusable subcomponents is particularly difficult because learners have to separate the relevant subcomponent (e.g., the specific pattern of knits and purls that make a rib knit) from details that are idiosyncratic to particular instances (e.g., the textures and techniques used in the rest of the sweater). We do not yet know to what extent pedagogical sampling models capture human behavior in this kind of teaching problem, where examples of the whole are used to teach parts.

To approach this question, we studied how people teach and learn motifs within a simple necklace‐building task inspired by prior studies of cultural transmission (Clegg & Legare, 2016b, 2016a; Kleiman‐Weiner et al., 2020). In Experiment 1 (*N* = 151), we tested how well existing pedagogical sampling models capture how teachers transmit motifs. In this task, teachers demonstrated motifs—here, patterns of beads that can be flexibly reused—by providing a single sample necklace. Our pedagogical sampling model provided a better quantitative fit to teachers' decisions than a baseline model that samples uniformly from all necklaces with the target motifs. It also captured an important qualitative pattern in teachers' behavior: teachers produce simpler examples than would be expected by chance. In Experiment 2 (*N* = 295), we tested the limits of existing models by giving human learners a single sample necklace and asking them to both infer the underlying motifs and produce two new necklaces that incorporate them. Learners performed better at both tasks when given examples generated by human teachers or sampled from the pedagogical sampling model, compared to examples generated by the baseline model. This effect was largely explained by simplicity—across the board, learners were better able to recover motifs when given simpler examples. However, learners performed best overall with human‐generated examples, suggesting that state‐of‐the‐art pedagogy models still miss aspects of what makes human teaching so effective. One key missing ingredient may be additional inductive biases that people bring to teaching, such as an expectation that learners will parse necklaces from left to right. Consistent with this idea, we found that a model that combines pedagogical sampling with a left‐to‐right bias provides the best fit to human behavior. By contrast, heuristic models that instantiate a left‐to‐right bias or a simplicity bias *without* pedagogical sampling did not fit teachers' decisions as well. We close by discussing how pedagogical sampling models could be extended further to better capture how abstractions are culturally transmitted. All experiment materials, data, and analysis code are publicly available at https://osf.io/rnb9e/?view_only=5fdd7fee414643748d40ee34cd7c8a8d.

## 2 Computational framework

### 2.1 Task setup

The experiments below use a necklace‐building task as a simple case study of how people acquire and transmit motifs. In prior work, necklace‐building tasks have been used to study basic mechanisms that drive cultural transmission, including how faithfully children imitate (Clegg & Legare, 2016b, 2016a) and how adults learn concepts from step‐by‐step demonstrations (Kleiman‐Weiner et al., 2020). Experimental stimuli were adapted from Kleiman‐Weiner et al. (2020).

**Fig. 1:**
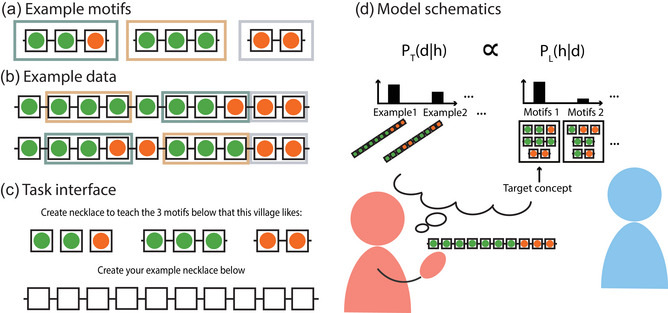
Teaching task. (a–c) Experiment interface: (a) On each trial, participants were shown the motifs favored by each village. (b) These motifs can be recombined to create many new necklaces. (c) Participants created a single sample necklace to teach learners these three motifs. (d) Model schematic: The pedagogical sampling model actively selects necklaces d to teach ($P_{T}\left(d|h\right))$) by anticipating how learners will recover the underlying motifs h from the sample necklace ($P_{L}\left(h|d\right))$.

In Experiment 1 (Teaching Abstractions), participants play the role of expert artisans; their task is to travel from one village to another, teaching an apprentice how to produce necklaces that will sell well in each village. In our setting, each necklace is a string of 10 orange and green beads that can be represented as a binary sequence. Each village has three favorite motifs, which are subsequences of two or three beads that can be recombined to make necklaces (Fig. 1a). A necklace sells well in a village if and only if it includes all three of the motifs favored in that village (Fig. 1b). In Experiment 2 (Learning Abstractions), participants played the role of the apprentice. In each village, participants saw one necklace generated by an expert teacher; their task was to infer the underlying motifs in the necklace and to use these motifs to create new necklaces of their own.

In both experiments, we modeled teachers' and learners' behavior using two models: the strong sampling model, which serves as a baseline and assumes that the teacher samples uniformly from all necklaces containing the correct motifs, and the pedagogical sampling model, which selects necklaces to maximize the learner's posterior beliefs in the correct motifs. We explain these models in more detail below.

### 2.2 Strong sampling model

Strong sampling refers to a process where examples are uniformly sampled from a target hypothesis (Shafto et al., 2014; Tenenbaum & Griffiths, 2001). In our task, each “example” is a sample necklace, and each “hypothesis” is a set of three motifs. In other words, the strong sampling model chooses uniformly among all necklaces that contain all three motifs favored by a particular village. We compared the strong sampling model to the behavior of both teachers and learners. In Experiment 1, comparing the fit of the pedagogical sampling model to that of the strong sampling model provides evidence about the extent to which teachers' decisions are guided by higher‐order inferences about a hypothetical learner's beliefs (see *Pedagogical sampling model*, below). In Experiment 2, we constructed a learner model that assumes that the teacher's examples were selected using strong sampling. We used this model as a baseline to measure how accurately human learners could be expected to recover the target hypothesis based on a single example (see *Baseline learner model*, below).

Given a hypothesis h, the strong sampling model predicts that the probability of selecting any sample necklace d is inversely proportional to the number of necklaces that contain the target motifs:

(1) $$P_{\text{strong}}\left(d|h\right)=\left\{\begin{array}{cc} \frac{1}{\left|h\right|} & \text{if}\;d\in h\\ 0 & \text{otherwise} \end{array}\right..$$

We generated the predictions of the strong sampling model by first creating a matrix C of hypotheses and data. The rows contain all possible 10‐bead necklaces ($2^{10}=1024$ possible necklaces) and the columns contain all possible triplets of motifs (with $2^{2}$ possible 2‐bead motifs and $2^{3}$ 3‐bead motifs, there are 22+233=220 possible hypotheses). Each cell $c_{d,h}$ of this matrix indicates whether the necklace d could have been generated by combining the three motifs in h ($c_{d,h}=1$) or not ($c_{d,h}=0$). Thus, Eq. 1 is equivalent to the following matrix operation:

(2) $$P_{\text{strong}}\left(d|h\right)=\frac{c_{d,h}}{\sum_{d^{\prime }}c_{d^{\prime },h}},$$

which corresponds to normalizing the entries of each column by its sum.

#### 2.2.1 Baseline learner model

The baseline learner model assumes that the teacher selects a sample necklace d using strong sampling. We can use Bayes' rule to obtain the posterior distribution over motifs given the example provided:

(3) $$P_{baseline}\left(h|d\right)=\frac{P_{strong}\left(d|h\right)P\left(h\right)}{\sum_{h^{\prime }}P_{strong}\left(d|h^{\prime }\right)P\left(h^{\prime }\right)},$$

where $P_{strong}\left(d|h\right)$ is the probability of selecting the sample necklace d under strong sampling when the true hypothesis is h (Eq. 1) and P(h) is the prior probability assigned to the hypothesis h. The model further assumes a uniform prior over all sets of motifs (h), so P(h) is the same for all h. Thus, Eq. 3 can be simplified as:

(4) $$P_{baseline}\left(h|d\right)=\frac{P_{strong}\left(d|h\right)}{\sum_{h^{\prime }}P_{strong}\left(d|h^{\prime }\right)}.$$

### 2.3 Pedagogical sampling model

The pedagogical sampling model chooses necklaces to teach by anticipating how the learner will recover motifs from the example provided. Thus, rather than sampling uniformly from all necklaces that contain the target motifs, this model favors necklaces that are consistent with fewer alternative hypotheses. Intuitively, this strategy reduces the risk that learners will recover the wrong set of motifs from the sample necklace.

As an illustration, suppose that a village favors necklaces that contain the motifs “000,” “11,” and “001.” These motifs can be used to make many necklaces, including the following two examples:

(5) $$\begin{array}{c} 0000000111\to [000]00[001][11]\\ 0001100011\to 0[001]1[000][11] \end{array}$$

Here, the left side of each line shows the necklace as it would appear to a learner, and the right side breaks down each example into its component motifs. Note that both of these examples are ambiguous; there are several alternative sets of motifs that could have generated either of these necklaces. However, the model favors the necklace “0000000111” because there are fewer incorrect ways to parse it. Besides the three target motifs, this necklace is consistent with four incorrect motifs (i.e., “111,” “011,” “01,” “00”), which make up 13 consistent but incorrect alternative hypotheses (e.g., “00|000|11,” “11|01|000”). By contrast, “0001100011” can be parsed incorrectly in more ways. In addition to the three correct motifs, this necklace is consistent with six incorrect motifs (i.e., “00,”“01,”“10,”“100,”“011,”“110”), which make up 46 consistent but incorrect alternative hypotheses (e.g., “00|100|11,” “110|011|000”).

More formally, this model characterizes pedagogy as a form of cooperative communication between a teacher and a learner (Shafto et al., 2021). The model assumes that both the teacher and the learner have common knowledge about a space of hypotheses (i.e., all possible triplets of motifs) and a space of data (i.e., all possible necklaces; Shafto et al., 2014). The teacher selects necklaces to show to the learner that will maximize the learner's beliefs in the true set of motifs favored by a particular village, and the learner works backwards from the necklace provided to infer what motifs the teacher is trying to demonstrate. These recursive inferences between the teacher and the learner are captured using the following system of equations:

(6) $$P_{\text{learner}}\left(h|d\right)=\frac{P_{\text{teacher}}\left(d|h\right)p\left(h\right)}{\sum_{h^{\prime }}P_{\text{teacher}}\left(d|h^{\prime }\right)p\left(h^{\prime }\right)},$$

(7) $$P_{\text{teacher}}\left(d|h\right)\propto P_{\text{learner}}{\left(h|d\right)}^{\alpha },$$

where α is a free parameter that controls how strongly the teacher favors examples that maximize the learner's posterior belief in the true motifs. In the results below, we fit α to individual participants' responses in Experiment 1.

Following the procedure in Shafto et al. (2014), we calculated a solution to this system of equations using fixed‐point iteration. We began by normalizing each column of the matrix C by its sum, as in the strong sampling model (Eq. 2). Next, we implemented the recursive inferences that distinguish pedagogical sampling from strong sampling by renormalizing this matrix. In the first iteration of this procedure, we normalized each row by its sum to generate $P_{\text{learner}}\left(h|d\right)$. Intuitively, each row of this matrix represents the posterior beliefs of a learner who assumes that the sample necklace d was generated using strong sampling. (Note that these first two iterations are equivalent to the strong sampling and learner models described in the previous section.) In the next iteration, we raised C to the power of α and normalized each column by its sum to generate $P_{\text{teacher}}\left(d|h\right)$. Each column of this matrix represents the choice probabilities of a teacher who selects examples by considering the beliefs of the learner in the prior iteration.

Each iteration of this procedure represents an additional recursive inference. For example, the next iteration yields the beliefs of a learner who tries to interpret what hypothesis the teacher is attempting to communicate, and the next iteration after that yields the choice probabilities of a teacher who tries to maximize the beliefs of a learner who actively interprets their examples, and so on. In principle, we could iterate this system of equations indefinitely; in practice, $P_{\text{learner}}\left(h|d\right)$ and $P_{\text{teacher}}\left(d|h\right)$ converge to a fixed point after a finite number of steps (Wang, Wang, Paranamana, & Shafto, 2020). We set the tolerance of convergence to $10^{-12}$.

### 2.4 Model variants

In addition to the baseline and pedagogical sampling models, we tested four model variants that incorporate inductive biases that teachers may use when selecting examples (see Supplementary Material, Section S2). Two of these variants extend the pedagogical sampling model: one adds a *left‐to‐right bias*, favoring examples where motifs appear sequentially from the leftmost bead; the other adds a *simplicity bias*, penalizing examples with higher algorithmic complexity. We also tested two heuristic models that apply these biases—left‐to‐right and simplicity—without pedagogical sampling, to test whether these biases alone can account for teachers' behavior.

## 3 Experiment 1: Teaching abstractions

In Experiment 1, participants played the role of teachers. Their task was to provide a single sample necklace to teach a learner generalizable “motifs” that could be recombined to produce new necklaces that would sell well in a particular village. We compared the necklaces generated by human teachers to those selected by a model that randomly selects a necklace that is consistent with the target motifs (strong sampling) and a model that aims to maximize the learner's posterior belief in the correct motifs (pedagogical sampling).

### 3.1 Methods

#### 3.1.1 Participants

We aimed to recruit 150 participants for the teaching task on Prolific (preregistration available at https://aspredicted.org/GPT_HNV). In both experiments, participants were recruited through the standard sample option; only participants who resided in the United States, had an approval rate over 95%, were fluent in English, and completed 100–10,000 studies were eligible to sign up. We obtained data from 151 participants (*M*(*SD*) age = 39.78(13.73), 94 female, 53 male, and 4 nonbinary), potentially due to a server error at the time of submission. Participants earned $3 for their participation, and they were told that they could earn a performance bonus of up to $1 based on how well participants in Experiment 2 learned from the examples they selected. Thus, participants were incentivized to provide helpful examples. In all experiments, participants provided informed consent in accordance with the requirements of the Institutional Review Board at Princeton University.

#### 3.1.2 Procedure

Participants played the role of master artisans; their task was to travel to 18 different villages and teach an apprentice how to produce necklaces that would sell well in each village. On each trial, participants were shown the motifs favored by a new village and were asked to create a single 10‐bead necklace that contains all motifs favored by that village. Each village had a unique set of motifs, and villages were presented in a randomized order.

On each trial, participants saw the three motifs favored by the village displayed on the top of the screen, and they typed in a sample necklace by placing beads on an empty string in sequence. For example, if a village had the motifs 000, *10*, **111** teachers could pass on these motifs by producing the necklace 000*10* **111** *10* (emphasis added for demonstration). Participants could erase beads from the sequence to correct mistakes and press a “submit” button when they were finished with the sample necklace. To better align the task with our modeling assumptions, we constrained participants' responses so that they had to include all three motifs in their sample necklace; if the necklace they produced was not valid, participants were prompted to correct the necklace before proceeding.

#### 3.1.3 Computational modeling

**Model fitting and comparison**: We compared models to participants' responses using random‐effects Bayesian model selection (Rigoux, Stephan, Friston, & Daunizeau, 2014). First, we used maximum likelihood estimation to fit the α parameter of the pedagogical sampling model to each participant's responses. For each participant, we then evaluated the fit of the strong sampling and the pedagogical sampling models using the Bayesian information criterion (BIC=klog(n)−2log(L)), where k is the number of free parameters in the model (0 for strong sampling and 1 for pedagogical sampling), n is the number of trials observed per participant (which was fixed at n=18), and L is the maximized value of the probability of the data under the model. Finally, we used −0.5×BIC as an estimate of log model evidence for each participant (Bishop & Nasrabadi, 2006) and used it to compute the protected exceedance probability (pxp) for each model. Protected exceedance probabilities treat models as random effects that can vary between participants; this measure can be interpreted as the probability that a given model occurs most frequently in the population.

**Model simulation**: In addition to the model comparison procedure described above, we also used fitted models to create simulated datasets. Intuitively, simulated datasets allow us to compare to what extent the necklaces produced by participants overlap with those that would be produced by each model. To create simulated datasets, we first used the fitted α parameters for each participant to create matrices of choice probabilities ($p_{T}\left(d|h\right)$) and then sampled from this matrix of choice probabilities to obtain simulated responses.

**Measuring sequence complexity**: We measured the algorithmic complexity of necklaces using the block decomposition method (Soler‐Toscano, Zenil, Delahaye, & Gauvrit, 2014; Zenil et al., 2018), as implemented in the *pyBDM* Python package (Talaga & Tsampourakis, 2024). Formally, algorithmic complexity refers to the length of the shortest computer program that can reproduce a given sequence. Computing this value exactly is computationally intractable for all but the shortest sequences. The block decomposition method approximates this value by dividing longer sequences into smaller fragments. Each fragment is then compared against a large lookup table of precomputed complexity values for short sequences. These local complexity estimates are combined—while accounting for repetition—to yield a complexity score for the full sequence.

We selected this measure of algorithmic complexity because it aligns closely with human judgments of simplicity (Zenil, Toscano, & Gauvrit, 2022). Sequences with lower algorithmic complexity tend to be judged as “simpler” by human observers, who are more likely to interpret them as the product of a regular, rule‐based process (e.g., alternating green and orange beads) rather than a random process (Griffiths & Tenenbaum, 2003). Lower‐complexity sequences also tend to be easier to remember, likely because they can be compressed into a more compact mental representation (Mathy et al., 2024; Planton et al., 2021). Fig. 2 shows necklaces that vary in algorithmic complexity.

**Fig. 2:**
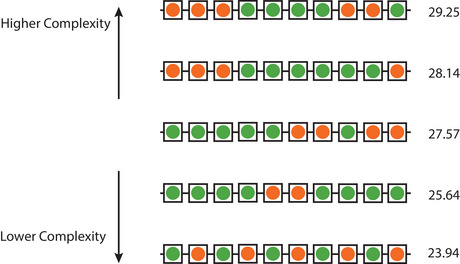
Necklaces of varying levels of algorithmic complexity. More complex necklaces are on top, while the simplest necklaces—those with the lowest algorithmic complexity scores—are on the bottom. Intuitively, the simplest necklaces can be described compactly using a regular process (e.g., “alternate green and orange beads”), while more complex necklaces require longer descriptions (e.g., “add three orange beads, then four green beads, then two orange beads, then one green bead”).

### 3.2 Results

Overall, participants' choices were better captured by the pedagogical sampling model than by the strong sampling model (pxp = 1.000; see Fig. 3c for model evidence). A closer look at the human‐generated and model‐simulated necklaces reveals that both human participants and the pedagogical sampling model tended to create sample necklaces that were simpler than those created by a strong sampling model (Fig. 3a,b). We performed a linear mixed‐effects regression that predicted the algorithmic complexity of sample necklaces based on fixed effects and random slopes of teacher type (human, pedagogical model, strong sampling model) and random intercepts by village (18 different ground‐truth sets of motifs). Both participants and the pedagogical sampling model created sample necklaces with lower algorithmic complexity than those produced by the strong sampling model (human‐generated vs. strong‐sampled necklaces: b=−0.565,t(17.001)=−6.709,p<.001; pedagogically sampled vs. strong‐sampled necklaces: b=−0.243,t(17.003)=−7.108,p<.001). These results suggest that the pedagogical sampling model captures a specific pattern in how people teach abstractions: Namely, effective teaching favors simpler examples. Note that we did not explicitly instruct the model to favor simpler examples—instead, this preference stems from a more general communicative principle.

**Fig. 3:**
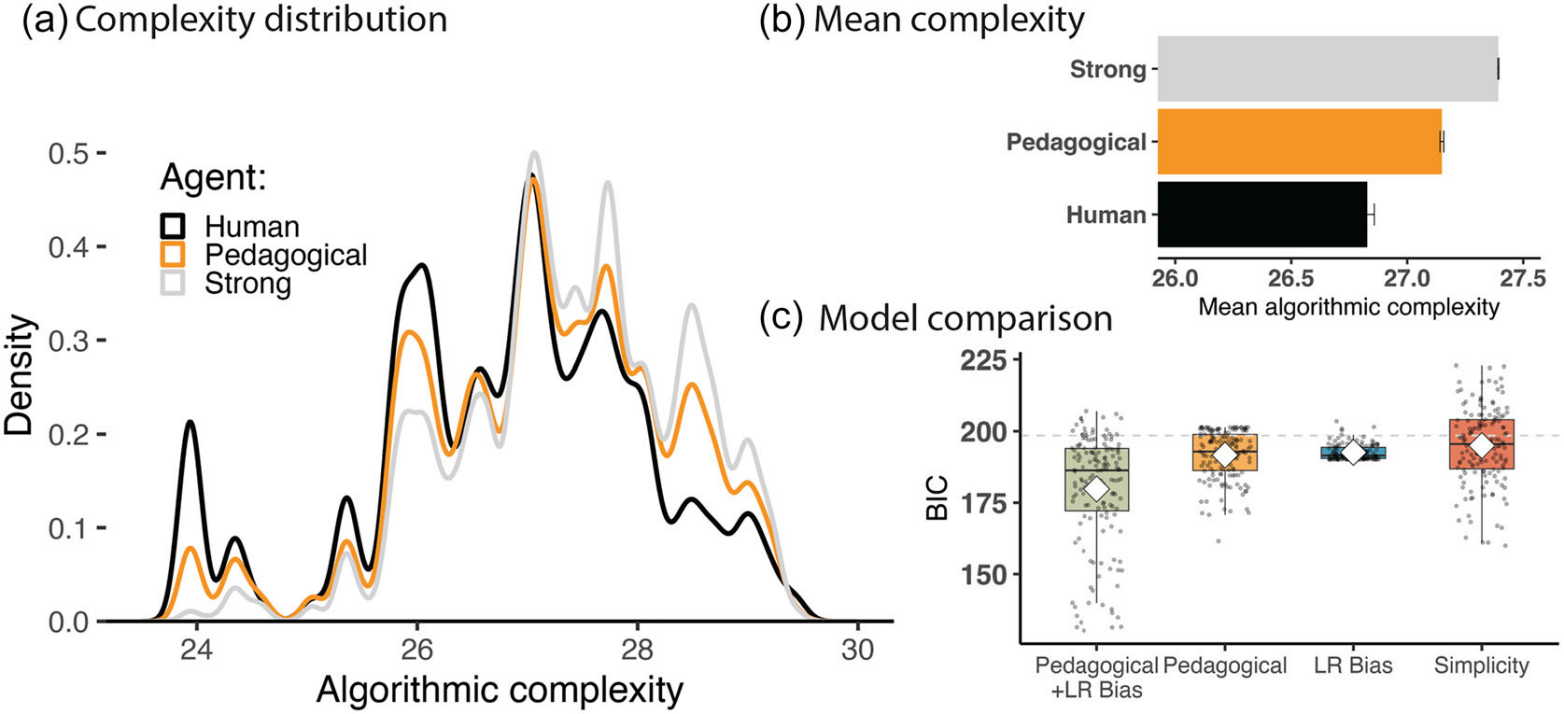
Experiment 1 results. (a) Distribution of algorithmic complexity scores for human‐generated necklaces (black line) and model‐simulated necklaces (gold, gray lines). (b) Average algorithmic complexity by teacher type. Human‐created sample necklaces have the lowest mean complexity. Error bars denote standard error of the mean. (c) Model comparison: Each dot represents the fit between model and a single participant's responses, as measured by the Bayesian information criterion (BIC). The gray dotted line indicates the BIC of the strong‐sampling model; because this model selects uniformly among all valid necklaces, the probability of each participant's response under this model is a fixed value. The box plots show BIC scores of the pedagogical sampling model (Pedagogical), the left‐to‐right bias model (LR Bias), and the model that combines both (Pedagogical + LR Bias), and the model based on the simplicity measure (Simplicity). Overall, participants' responses were better captured by the pedagogical sampling model combined with the left‐to‐right bias.

However, pedagogical sampling alone does not account for the full pattern of participants' responses. The examples generated by participants were still simpler than those simulated by the pedagogical sampling model (pedagogically sampled vs. human examples: b = 0.322, t(16.997)=5.648, p<.001). For example, when trying to teach the motifs 00, *100*, **101**, participants were more likely to teach the necklace “00*100* **101**00,” while the pedagogical sampling model would assign a higher probability to teach “*100*0000**101**.” The necklace favored by human teachers has a lower algorithmic complexity score than the one favored by the pedagogical sampling model (human‐generated necklace: 26.99, model‐generated necklace: 27.69). Thus, one possible explanation for this discrepancy is that human teachers may have additional reasons to prefer simpler necklaces, beyond considering the learner's beliefs. For example, teachers may prefer simple examples because they are easier to produce, or because they are more aesthetically pleasing. We tested two alternative models that speak against this possibility. First, adding a complexity penalty to the pedagogical sampling model did not fully close this gap (Fig. S1). Second, the standard pedagogical sampling model outperforms a “simplicity” model that favors simpler examples *without* reasoning about the learner's mental states (Fig. 3c). These results suggest that participants do not favor simplicity for its own sake; instead, participants' decisions may have been guided by additional inductive biases about what kinds of simple examples are useful to human learners.

Another possibility is that teachers may have expectations about how learners will *interpret* the examples that are not captured by the standard pedagogical sampling model. In particular, the sample necklace favored by human teachers is structured so that the motifs can be read from left to right, like a script: each motif is first presented once, with no extraneous beads between them (“00*100* **101**…”). By contrast, the example favored by the pedagogical sampling model does not have this property; instead, one motif is repeated midway through the necklace, so the motifs cannot be read out consecutively (*100*00…**101**). To test whether human‐generated necklaces consistently show this property, we implemented a variant of the pedagogical sampling model with a left‐to‐right bias; this model favors example necklaces where the three motifs appear one after another, starting from the leftmost bead. Indeed, the pedagogical model with a left‐to‐right bias better captures participants' behavior than either the standard pedagogical sampling model or a heuristic model with the left‐to‐right bias alone (pxp = 1.000; see Fig. 3c). This result suggests that human teachers may expect learners to parse necklaces directionally.

Put together, our results suggest that teachers transmit motifs by selecting simple examples. Our model comparisons reveal that this pattern of behavior cannot be explained by simple heuristics that value simplicity for simplicity's sake, but that it instead arises from more general computational principles of teaching. However, we also find evidence that participants' decisions may have been guided by additional inductive biases about what kinds of simple examples are useful to learners. If this is the case, then learners might derive benefits from human‐generated examples that are not fully captured by pedagogical sampling alone. In the following experiment, we tested whether learners can indeed recover motifs from a single sample necklace, and whether they learn best from examples generated by human teachers.

## 4 Experiment 2: Learning abstractions

Our results thus far suggest that reasoning about learners' mental states drives teachers to create simpler necklaces than would be expected by chance. However, it is an open question whether this simplicity aids learning—that is, whether the examples selected by teachers actually help others recover the underlying motifs. In our second experiment, we approached these questions by directly testing how well people learn triplets of motifs from observing a single sample necklace. Sample necklaces were selected from those generated by human teachers in Experiment 1 and from simulated datasets generated using the strong sampling and pedagogical sampling models.

### 4.1 Methods

#### 4.1.1 Participants

We aimed to recruit 300 participants for the learning task on the Prolific platform using the standard sample option (https://aspredicted.org/CK3_1NP). We lost data from five participants due to server errors, leaving us with 295 participants (*M*(*SD*) age = 40.02(12.31), 142 female, 149 male, and 4 nonbinary). Participants were paid $5 for completing the task, plus a bonus of up to $1 contingent on performance.

#### 4.1.2 Procedure

Participants were told that they were apprentices to a master artisan. Their task was to travel to 18 villages and learn how to produce necklaces that would sell well in each village. As in Experiment 1, participants were told that necklaces would only sell well in a particular village if and only if they contained all three motifs favored by that village. However, participants in Experiment 2 were not shown these motifs directly; instead, they saw a single sample necklace generated by a teacher and had to infer the motifs represented by the necklace.

On each trial, participants saw the sample necklace on the top of the screen and answered two questions about the village's motifs (Fig. 4a). First, participants typed the motifs that they believed that the village favors. As described above (*Computational framework*), each motif was a sequence of two or three beads. Participants could erase the beads that they typed to correct mistakes, and submit the motifs when they were ready. Once they submitted the three motifs, participants could not change their responses. Next, participants were shown two empty necklaces and asked to create two new necklaces that would sell well in that village. Participants could only proceed if they produced two unique, length‐10 necklaces that were distinct from the sample necklace. Villages were presented in a random order.

**Fig. 4:**
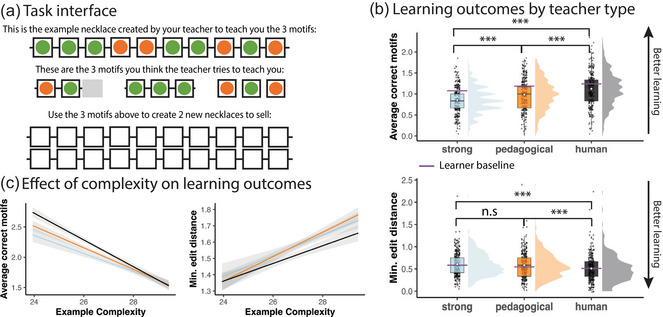
Experiment 2 results. (a) Task interface: Participants saw a single sample necklace (top) that contained all three motifs favored by that village. They then explicitly reported the motifs (middle) and created two unique necklaces that were distinct from the sample necklace (bottom). (b) Learning outcomes by teacher type: We measured learners' performance based on the number of motifs that they correctly reported (range 1–3; higher scores indicate better performance) and the minimum number of edits needed to transform learners' necklaces into a correct necklace (range 0–10; lower scores indicate better performance). Each point denotes a single participant's average performance; white diamonds denote average scores by teacher type. As a baseline, we compared these scores to the performance of a learner model that infers the underlying motifs by assuming that teachers select examples using strong sampling (purple line). (c) Correlation between the algorithmic complexity of the sample necklace and learner performance, as indexed by the number of correct motifs (left) and edit distance (right). Shaded areas denote 95% confidence intervals. Participants performed better when they were shown simpler sample necklaces.

Participants completed three within‐subjects conditions, which differed in how sample necklaces were generated. In the *Human* condition, participants saw sample necklaces created by participants in Experiment 1. By contrast, in the *Pedagogical* and *Strong* conditions, participants were shown sample necklaces that were simulated using the pedagogical‐ and strong‐sampling models, respectively. (See Experiment 1 for more details on how model‐simulated necklaces were generated.) Participants completed 18 trials total, comprising six trials for each teacher type that were presented in a randomized, interleaved order. Participants were blind to condition; that is, they did not know what type of teacher generated each necklace. This experimental manipulation allows us to compare the effectiveness of human‐ and model‐generated examples.

### 4.2 Results

We measured participants' performance using two outcome measures: The number of unique motifs correctly reported by the participant (*correct motifs*; range: 0–3, where higher scores indicate better performance) and the minimum number of changes that would have to be made to change participants' necklaces into a necklace that is consistent with the ground‐truth motifs (*minimum edit distance*; range: 0–10, where lower scores indicate better performance). We modeled each outcome using a mixed‐effects ordinal regression with fixed effects of teacher type (i.e., Human, Pedagogical, Strong, with Strong as the reference level) and random slopes of teacher type by village.

Overall, participants learned best when they received sample necklaces selected by human teachers, rather than necklaces generated by the pedagogical‐ and strong‐sampling models (Fig. 4b). Participants explicitly reported more correct motifs when the examples were generated by the pedagogical‐sampling model (effect of Pedagogical vs. Strong examples on correct motifs: b=0.349 ,z=5.120, p<.001) and by human participants from Experiment 1 (Human vs. Strong: b=0.702, z=8.011, p<.001), compared to the strong sampling model. However, participants recovered the most motifs overall when they received examples generated by a human (Human vs. Pedagogical: b=0.341,z=4.230,p<.001). We find similar results when examining how close learners were to generating a valid necklace of their own. Participants generated necklaces that were closer to the target motifs (i.e., had lower minimum edit distances) when they received sample necklaces from a human teacher (effect of Human vs. Strong examples on minimum edit distance: b=−0.336,z=−4.249,p<.001), but not necklaces generated by the pedagogical‐sampling model (Pedagogical vs. Strong: b=−0.091,z=−1.090,p=.276). Overall, participants produced more accurate necklaces when they received examples from a human (Human vs. Pedagogical: b=−0.244,z=−4.354,p<.001).

On average, participants recovered approximately one of the three motifs specific to each village (mean(SE) number of motifs: 0.987(0.015)) and produced necklaces that were less than one bead away from an acceptable necklace (mean(SE) minimum edit distance: 0.566(0.013)) when provided a single sample necklace by a teacher. How well could participants be expected to do, given the sparse and ambiguous information given to them? We benchmarked participants' performance against the performance of the baseline learner model described above. We used this model in two ways. First, we sampled a triplet of motifs from $P_{baseline}\left(h|d\right)$ (Eq. 4) to model how learners reported the motifs contained within each sample necklace. Second, the model samples uniformly from all necklaces that contain this triplet to create two new necklaces.

Overall, we find that the learner baseline captures qualitative patterns in learner performance. Like human learners, the baseline learner recovered approximately one of the three motifs specific to each village (mean(SE) number of motifs: 1.168(0.004)) and produced necklaces that were less than one bead away from an acceptable necklace (mean(SE) minimum edit distance: 0.547(0.006)). To compare quantitative fits, we compared participants' actual performance against this benchmark using paired Wilcoxon tests. Regardless of teacher type, participants reported slightly *fewer* correct motifs than the learner baseline (all p<.05 with the average difference of 0.178 motifs) and produced necklaces with *similar* minimum edit distances as the learner baseline (all p>.2). We note one difference between the learner baseline and participants' behavior: In 30% of trials, participants reported motifs that were not consistent with the sample necklace they were provided. Thus, it is possible that participants may have been inattentive. In an exploratory analysis, we found that excluding these observations improved human learners' average performance slightly but did not affect our interpretation of the results (see Supplementary Material). Thus, while participants underperformed slightly relative to our baseline, they recovered about as much information as we could expect from a single example.

To understand what makes human‐generated examples particularly effective, we next used mixed‐effects ordinal regressions to model learners' performance based on an interaction between teacher type and the algorithmic complexity of the sample necklaces provided; we also included random effects of teacher type and algorithmic complexity by village. Participants who received more complex sample necklaces reported fewer correct motifs (effect on correct motifs: b=−0.293,z=−3.699,p<.001) and produced necklaces that were farther from correct necklaces (effect on minimum edit distance: b=0.269,z=4.721,p<.001).

Together, our results suggest that simplicity is beneficial: Participants indeed learned better when they were given simpler examples. However, participants still learned best when given examples by human teachers—even though they were not aware of our experimental manipulation—which suggests that existing models of teaching do not fully capture what makes human teaching so effective. In the Discussion, we will consider how to bridge this gap.

## 5 Discussion

Teaching useful abstractions underlies cultural and technological achievements that require flexibility, innovation, and creativity. In this paper, we tested to what extent existing models of teaching capture how humans teach motifs—design patterns that can be flexibly recombined to create new objects. Overall, we found that the general computational principles that underlie effective teaching and communication, as formalized by the pedagogical sampling model, also capture key patterns in how humans teach and acquire recombinable motifs. Specifically, teachers favor simple examples, and learners learn best from simple examples, without explicitly building simplicity as an assumption into our models of either teachers or learners. However, our results also suggest that human teachers and learners are even more sensitive to simplicity than standard pedagogical sampling models would predict, highlighting an exciting direction for future research.

Our results speak to theoretical debates on the importance of providing simple data to teach complex concepts. Prior work in machine learning has argued that providing simpler examples at the beginning of training can speed learning in a variety of domains, such as providing shorter sentences to teach grammars (Elman, 1993), simpler shapes to aid visual classification (Bengio, Louradour, Collobert, & Weston, 2009), or easier motor tasks to aid skill learning (Karpathy & Van de Panne, 2012). While prior work has used lengthy training periods with plenty of examples, our task—where teachers strategically select a single example—provides insights into *when* and *why* simple examples aid learning (see also Rafferty & Griffiths, 2010; Zhao, Lucas, & Bramley, 2024). Our model comparison suggests that teachers do not favor simplicity for its own sake; a heuristic model that selects examples in proportion to their simplicity does a poor job of capturing teachers' examples. Instead, teachers strategically create simple examples that constrain learners' interpretations. Intriguingly, we found that human teachers produced examples that were *even simpler* than the pedagogical sampling model would predict, and that learners benefited the most from these examples. This pattern of results suggests that teachers' decisions may be driven by additional considerations that are not fully captured by standard pedagogical sampling models. As initial evidence for this idea, we also find that teachers expect learners to parse necklaces directionally, starting from the leftmost bead.

Our findings also contribute to a growing literature on the interplay between social and physical reasoning (Jara‐Ettinger & Schachner, 2024; Liu, Outa, & Akbiyik, 2024). Much of this work has focused on how observers extract social meaning from physical artifacts. For instance, people can infer that a seat is taken because a jacket is draped over it (Lopez‐Brau & Jara‐Ettinger, 2023), or reconstruct chains of social transmission from recurring design features (Pesowski et al., 2020; Schachner, Brady, Oro, & Lee, 2018). These studies emphasize that physical artifacts carry traces of the intentions, goals, and social histories of their creators. Our work extends this perspective by examining how social reasoning shapes the *construction* of physical artifacts. In our task, teachers deliberately embedded motifs within a necklace so that learners would discover them. This required anticipating how learners would mentally decompose the sequence into meaningful fragments. (In this respect, teachers' inferences have interesting parallels to how we computed algorithmic complexity—by breaking down longer sequences into smaller, structured parts.) In this way, our findings suggest that social reasoning guides not only how people interpret physical artifacts, but also how they construct artifacts to make their intended structure recognizable to others.

Overall, our findings shed light on the promise and limitations of using Bayesian models of pedagogy to understand how humans transmit abstract knowledge. However, there are several aspects of this domain that our simple task and model do not capture. Most notably, our task limits teachers to providing a single example. This constraint provides a clearer picture what teachers value, and it reveals that learners can partially recover motifs from very sparse data. However, this constraint also obscures the ways that learners may refine abstract concepts over longer interactions. Novices do not learn how to knit a moss stitch or play musical chords from a single example, but instead through repeated interactions where an expert provides opportunities for learners to observe skills, corrects their work, and sometimes provides explicit instruction (Kline, 2015). Thus, one important direction for future work is to examine how teachers develop longer curricula to refine learning, such as by providing contrasting cases (Gentner & Hoyos, 2017; Gick & Holyoak, 1983) or by incorporating opportunities for learners to generate their own examples (Chen et al., 2024; Schwartz, Chase, Oppezzo, & Chin, 2011).

In addition, while the motifs in our task were simple sequences that could be recombined in arbitrary ways, real‐world cultural motifs are often imbued with meaning (Cohn, 2012; Hawkins, Sano, Goodman, & Fan, 2023; Long, Fan, Huey, Chai, & Frank, 2024). For example, skull motifs can remind viewers of the inevitability of death, and peonies appear frequently in Chinese art as a symbol of prosperity and wealth. It is an open question how teachers and learners coordinate on the meaning of motifs, or how these meanings constrain how motifs are deployed. Thus, more work is needed to extend existing theories of pedagogy to fully embrace the social meaning of motifs.

Beyond passing down a collection of facts about the world, teaching imparts “tools for thought” that can empower learners to make new discoveries and create new things. Our work provides a theoretical and empirical framework to understand how teachers transmit reusable knowledge. We hope that revealing further components of this ability will provide a fuller picture of how human intelligence is augmented by social learning and culture.

## Supporting information

Data S1
