# Supplementary material for: Teaching Recombinable Motifs Through Simple Examples
Source: Cogn Sci. 2025 Aug 14;49(8):e70103. doi: 10.1111/cogs.70103 (PMC12351526; doi:10.1111/cogs.70103)
Supplement: Supplementary file 1 — Data S1 [file COGS-49-e70103-s001.zip › cogs70103-sup-0001-SuppMat/motifs_supplement_final.pdf]

Supplemental Material

July 24, 2025

1 Stimuli

We chose 18 triplets of motifs for the 18 villages that participants visit in both the teacher task (Experiment 1) and learner task (Experiment 2). 9 villages favor triplets of motifs that contain 2 motifs with 2 beads and 1 motif with 3 beads, while the remaining villages favor triplets of motifs that contain 1 motif with 2 beads and 2 motifs with 3 beads. We avoided triplets of motifs that contain either all length-2 or length-3 motifs because the pedagogical sampling and strong sampling model do not make clearly distinguishable predictions for this subset of stimuli. The stimuli are: “10|010|011", “01|011|101", “01|10|010", “00|11|010", “00|01|110" ,“01|11|101", “10|11|110", “00|011|100", “11|010|101" ,“01|101|110",“00|10|011", “10|010|101", “11|001|011", “01|11|010", “01|10|101" , “00|100|101", “00|01|010", “01|010|101". “1” represents orange beads and “0” represents green beads.

2 Experiment 1: Variants of the pedagogical sampling model

2.1 Building a complexity penalty into the pedagogical sampling model

In the main text, we report that teachers select examples that are even simpler than those selected by the pedagogical sampling model. As an exploratory analysis, we attempted to bridge this gap by extending the model with a penalty for more complex examples.

We first obtained the learner's posterior beliefs from the pedagogical sampling model, as described in the main text:

$$P_{\text{learner}}(h|d) = \frac{P_{\text{teacher}}(d|h)p(h)}{\sum_{h'} P_{\text{teacher}}(d|h')p(h')}, \tag{1}$$

Next, we defined the utility of teaching hypothesis h (a triplet of motifs) with example d (a sample necklace) by combining the learner's posterior beliefs with the simplicity score:

$$U(d|h) = ln(P_{\text{learner}}(h|d)) - wC(d) \tag{2}$$

where the negative surprisal term, $ln(P_{\text{learner}}(h|d))$, captures the informational value of the example to the learner, and the complexity penalty, $C(d)$, is defined as the algorithmic

complexity of d. w is the weight of the complexity penalty. Lastly, the utility score is converted into the probability of choosing d through a softmax function:

$$P(d|h) = \frac{e^{U(d|h)}}{\sum_{d'} e^{U(d'|h)}} \tag{3}$$

We used the model estimation and model comparison procedures described in Experiment 1 of the main text. First, we used maximum likelihood estimation to fit the α and w parameters of the model. Next, we used Bayesian model selection to compare the fit of complexity penalty model (Figure S1, "with simplicity") to the original pedagogical sampling model (Figure S1, "without simplicity"). Even after directly penalizing complex examples, the original pedagogical sampling model best captures the behavior of human teachers (pxp = 1). These results suggest that people may not favor simpler examples for simplicity's sake; instead, the decisions of human teachers may be guided by additional inductive biases that are not captured by existing theories.

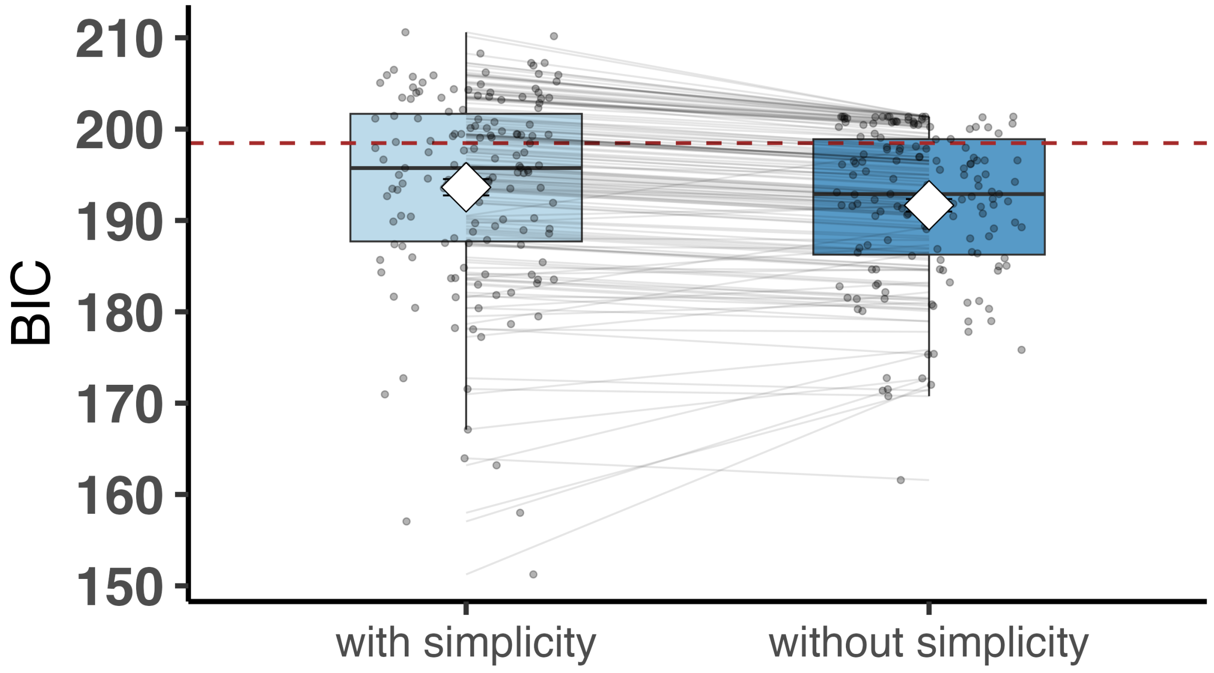

Figure S1: Bayesian information criterion (BIC): each dot represents the BIC of each model for each participant, where lower values indicate better model fit. The red dotted line indicates the BIC of the strong-sampling model for all participants. Overall, adding a complexity penalty (with simplicity) does not improve model fit relative to the standard pedagogical sampling model (without simplicity).

2.2 Baseline simplicity model

In Figure 2 of the main text, we also reported performance of a baseline simplicity model that predicted teacher's choices using only the simplicity of the necklaces. Under this model, the utility of an example necklace is simply its negative algorithmic complexity:

$$U(d|h) = -C(d) \tag{4}$$

The utility score is converted into the probability of choosing d using a softmax function (Equation 3). Note that this model does not depend on the hypothesis h and has no free parameter.

2.3 Left-to-right bias model

Another possible explanation for the discrepancy between human- and model-generated examples is that teachers may have additional expectations about how learners will interpret the examples. In the main text, we test this idea by implementing a variant of the pedagogical sampling model that expects that learners will parse examples directionally, starting from the leftmost bead. To formalize this "left-to-right bias", we ranked necklaces based on how early motifs are added to the necklace. For example, suppose that a teacher is asked to convey the motifs **10**, *11*, and 001. The necklaces that are best aligned with a left-to-right bias are those that place a motif on the leftmost bead and that have no beads between motifs, such as **10***11*001100. By contrast, necklaces such as **10**001100*11* would have a lower rank because there are extraneous beads dividing the second and third motif.

To be more precise, we arranged all necklaces that were consistent with a triplet of motifs based on the distance between the start of the sequence and the start of the first motif, then based on the distance between the end of the first motif and the start of the second motif, and so on. Ranks ranged from 1 to 35; larger numbers indicate that motifs appeared closer to the leftmost bead of the sequence. In the example above, **10***11*001100 would have a higher score than, e.g.,100**10***11*001, and necklaces with the same number of extraneous beads would receive the same score. To convert these rankings into a left-to-right bias score ($B(d|h)$), we divided ranks by the highest possible rank. Thus, $B(d|h)$ takes on values between 0 and 1, where higher values indicate closer alignment with a left-to-right bias.

Under this model, the utility of an example necklace is simply its left-to-right bias score:

$$U(d|h) = B(d|h) \tag{5}$$

The utility score is converted into the probability of choosing d using a softmax function (Equation 3).

2.4 Building a left-to-right bias into the pedagogical sampling model

As in Equation 2, the left-to-right bias score was combined with a measure of informational value to compute the utility of teaching necklace d:

$$U(d|h) = ln(P_{\text{learner}}(h|d)) + wB(h|d) \tag{6}$$

where w is the weight of the left-to-right bias. Lastly, the utility score is converted into the probability of choosing d using a softmax function (Equation 3).

3 Comparing learner performance to the baseline learner model after exclusions

In the main text, we note that human learners sometimes reported motifs that were inconsistent with the sample necklace they had received. It is possible that these trials reflect instances where participants were inattentive or made typing errors. Therefore, as an exploratory analysis, we also compared human learners' performance to the baseline learner model after excluding these trials. After excluding the 30% of the trials where the inferred motifs were not consistent with the example necklace, we overall saw a slight improvement in learners' performance compared to the learner baseline model. On average, participants recovered approximately one of the three motifs specific to each village (mean(SE) number of motifs: 1.091(0.015)) and produced necklaces that were less than one bead away from an acceptable necklace (mean(SE) minimum edit distance: 0.506(0.011)). The baseline learner recovered approximately one of the three motifs specific to each village (mean(SE) number of motifs: 1.163(0.007)) and produced necklaces that were less than one bead away from an acceptable necklace (mean(SE) minimum edit distance: 0.550(0.009)). After exclusion, participants still reported *fewer* correct motifs than the learner baseline if the examples were chosen by the models (all $p < .05$). However, if examples came from human teachers,

participants reported a *similar amount* of correct motifs as the baseline learner model ($p = .80$). Participants also produced necklaces with *similar* minimum edit distances as the learner baseline if the examples came from the pedagogical sampling model ($p = .285$). However, if the examples came from the strong sampling model or human teachers, participants produced necklaces with *smaller* minimum edit distances than the learner baseline (all $p < .01$).
